# *Orientia tsutsugamushi* alters the intranuclear balance of cullin-1 and c-MYC to inhibit apoptosis

**DOI:** 10.1128/iai.00559-24

**Published:** 2025-02-20

**Authors:** Paige E. Allen, Haley E. Adcox, Thomas E. Siff, Sarika Gupta, Jason R. Hunt, Jason A. Carlyon

**Affiliations:** 1 Department of Microbiology and Immunology, Virginia Commonwealth University Medical Center, School of Medicine 542826 , Richmond, Virginia, USA; University of California Davis, Davis, California, USA

**Keywords:** *Orientia tsutsugamushi*, scrub typhus, obligate intracellular bacterium, *Rickettsia*, cullin-1, ankyrin repeat, Ank, c-MYC, SCF, apoptosis

## Abstract

Cullin-1 (Cul1), a cullin–RING ubiquitin ligase component, represses c-MYC activity in the nucleus. *Orientia tsutsugamushi* causes the potentially fatal rickettsiosis, scrub typhus. The obligate intracellular bacterium encodes an arsenal of ankyrin repeat-containing effectors (Anks), many of which carry a eukaryotic-like F-box motif that binds Cul1. *O. tsutsugamushi* reduces Cul1 levels in the nucleus. This phenomenon is not due to an alteration in Cul1 neddylation but is bacterial burden- and protein synthesis-dependent. Five of the 11 Anks capable of binding Cul1 (Ank1, Ank5, Ank6, Ank9, Ank17) sequester it in the cytoplasm when each is ectopically expressed. Ank1 and Ank6 proteins with alanine substitutions in their F-boxes that render them unable to bind Cul1 cannot exclude Cul1 from the nucleus. Coincident with the reduction of Cul1 in the nuclei of *Orientia*-infected cells, c-MYC nuclear levels are elevated, and Cul1 target genes are differentially expressed. Several of these genes regulate apoptosis. The resistance of *O. tsutsugamushi*-infected cells to staurosporine-induced apoptosis is recapitulated in uninfected cells expressing Ank1 or Ank6 but not alanine-substituted versions thereof that cannot bind Cul1. Other F-box-containing Anks that cannot bind or exclude Cul1 from the nucleus also fail to confer resistance to apoptosis. Overall, *O. tsutsugamushi* modulates the Cul1:c-MYC intranuclear balance as an anti-apoptotic strategy that is functionally linked to a subset of its F-box-containing Anks.

## INTRODUCTION

*Orientia tsutsugamushi* is a mite-transmitted obligate intracellular bacterium that causes scrub typhus (1). *O. tsutsugamushi* invades leukocytes and disseminates via the lymphatics to invade endothelial cells of major organs (2). Acute scrub typhus symptoms include fever, headache, chills, arthralgias, myalgias, lymphadenopathy, and a maculopapular rash due to blood leakage from a damaged endothelium. In the absence or delay of antibiotic therapy, the disease can progress to respiratory distress, pneumonitis, meningitis, systemic vascular collapse, shock, multiorgan failure, and death (3–5). Non-travel related cases of scrub typhus, as well as serologic and molecular detection of *Orientia* DNA in the United Arab Emirates, several African countries, Peru, Chile, and the United States, signify the disease as an emerging global health threat (6–13).

The *O. tsutsugamushi*–host cell interactions that influence scrub typhus pathobiology and disease outcome are inadequately defined. A platform for gaining insight into how this genetically intractable microbe modulates eukaryotic processes lies in studying its repertoire of ankyrin repeat (AR)-containing effectors (Anks). The AR is a highly conserved protein–protein interaction motif (14), and the acquisition of genes encoding AR proteins is linked to enhanced virulence of intracellular pathogens (15, 16). *O. tsutsugamushi* expression of Anks during infection of tissue culture cells and in scrub typhus patients has been confirmed (17–22). Most *O. tsutsugamushi* Anks exhibit a bipartite architecture consisting of N-terminal tandemly arrayed ARs and a C-terminal F-box that exists independently or as part of a pox protein repeats of ankyrin C-terminal (PRANC) domain (23, 24). The *O. tsutsugamushi* F-box is homologous to and functionally mimics the mammalian F-box, which is the substrate recognition motif of the Skp, cullin, F-box-containing (SCF) E3 ubiquitin ligase complex that ubiquitinates proteins for 26S proteasomal degradation (23, 25, 26). The SCF complex quaternary structure consists of S-phase kinase-associated protein 1 (Skp1), cullin-1 (Cul1), RING (really interesting new gene) box 1 (Rbx1), and one of at least 78 F-box-containing proteins (FBPs). Cul1 acts as a scaffold that interacts with Skp1 and Rbx1 (26, 27). The FBP associates with Skp1 and Cul1 while also binding a target substrate using a separate protein–protein interaction domain (23, 26, 28, 29). The Cul1–Rbx1 catalytic core recruits an E2 enzyme that ubiquitinates the FBP-bound substrate (26). In addition to *O. tsutsugamushi*, other intracellular microbes encode F-box-containing Anks that are critical for fitness and pathogenesis (24, 25, 30–33). The working model is that the AR domain binds a host cell protein, while the F-box nucleates the SCF complex to mediate ubiquitination and degradation of the bound target (20, 23–25, 30, 32–34). This model has been validated for *O. tsutsugamushi* Ank5, which directs SCF-dependent ubiquitination and 26S proteasomal degradation of the major histocompatibility class I gene transactivator, NLRC5 (NOD-, LRR-, and CARD-containing 5/class I transactivator) (22).

Analysis of eight *O. tsutsugamushi* strain genomes found that the majority of their encoded Anks carry an F-box (35). Functions have been ascribed to a few *O. tsutsugamushi* Anks based on their ability to reproduce infection-associated phenomena when ectopically expressed, including manipulation of immune responses, secretory pathways, and transcription. Most of these modulatory functions require the F-box (17, 18, 20, 22, 36). When *O. tsutsugamushi* Anks were assessed for toxicity in yeast, 11 of the 12 that carry F-boxes were toxic, and 10 of these either lost or exhibited reduced toxicity when the F-box was removed. Only two of the seven Anks that lack F-boxes were toxic (25). Thus, the ability of most *O. tsutsugamushi* Anks to manipulate host cell processes is F-box-dependent.

Mammalian cells express eight cullin isoforms (37). While certain cullins localize to the nucleus and others are excluded, Cul1 exists in the cytoplasm and nucleus. It is one of only two cullins that associate with DNA at E-box sites. The E-box is a DNA regulatory motif in promoter and enhancer regions. Cul1 represses the function of a well-known E-box binding protein, c-MYC, to inhibit expression of a subset of target genes (38). c-MYC influences expression of 10 to 15% of human genes associated with a range of biological functions, including apoptosis and cell cycle progression (39). Cul1 is postulated to act as a transcriptional repressor by mediating site-selective proteasomal degradation of c-MYC bound at specific promoters (38). Given its arsenal of Ank-F-box effectors that coopt Cul1, *O. tsutsugamushi* has the potential capacity to modulate gene expression by altering Cul1 and c-MYC nuclear levels.

Here, we demonstrate that *O. tsutsugamushi* inhibits Cul1 accumulation in the nucleus. This phenotype can be copied by a subset of ectopically expressed F-box-containing Anks in a Cul1 binding-dependent manner. c-MYC nuclear levels are elevated during *Orientia* infection, and Cul1-regulated genes, several of which are associated with apoptosis, are differentially expressed. The resistance of *O. tsutsugamushi* infected cells to apoptosis is recapitulated by uninfected cells ectopically expressing Anks that impair Cul1 nuclear accumulation. Overall, we describe a mechanism by which *O. tsutsugamushi* counters innate defenses and links several of its Anks to this promicrobial strategy.

## RESULTS

### *O. tsutsugamushi* reduces Cul1 levels in the nucleus

**Fig 1:**
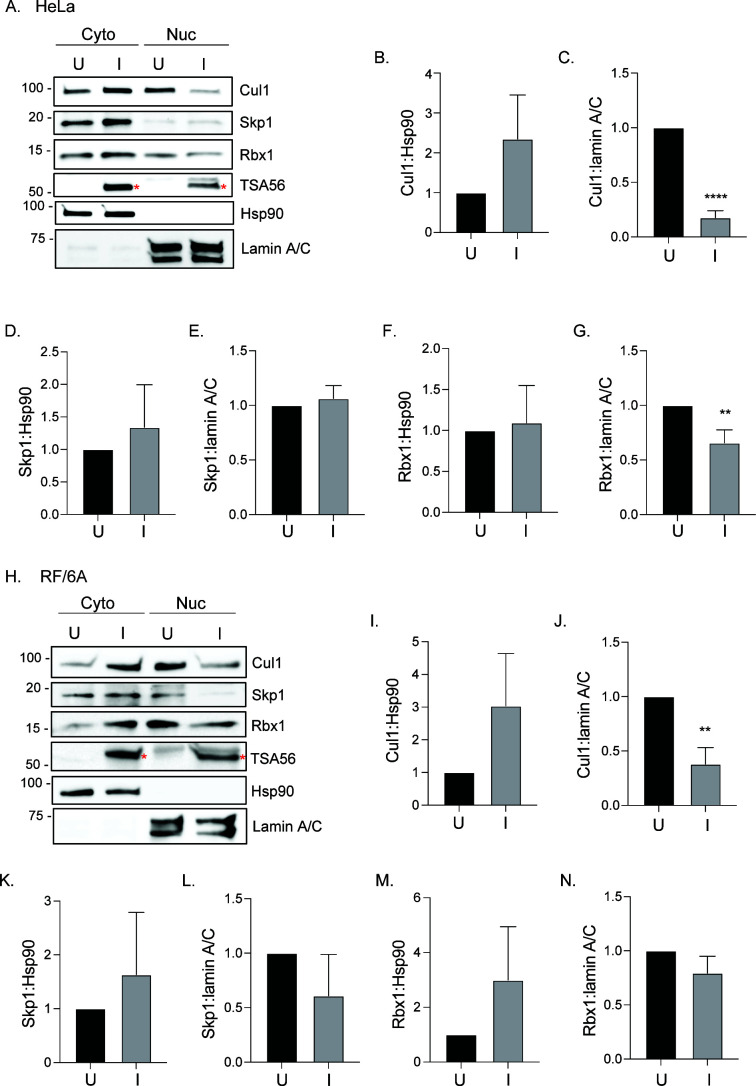
*O. tsutsugamushi* impairs Cul1 accumulation in the nucleus. HeLa cells (A to G) or RF/6A cells (H to N) were either mock [U] or infected [I] with *O. tsutsugamushi* at an MOI of 10, lysed, and separated into cytoplasmic [Cyto] and nuclear [Nuc] fractions. At 72 h, fraction lysates were subjected to western blot analyses using antibodies against Cul1, Skp1, and Rbx1 to confirm their presence, Hsp90 and lamin A/C to assess fraction purity, and TSA56 to confirm *O. tsutsugamushi* infection (A and H). Red asterisks denote TSA56 bands. Ratios of the densitometric values normalized to the uninfected control for Cyto Cul1:Cyto Hsp90 (B and I), Nuc Cul1:Nuc lamin A/C (C and J), Cyto Skp1:Cyto Hsp90 (D and K), Nuc Skp1:Nuc lamin A/C (E and L), Cyto Rbx1:Cyto Hsp90 (F and M), and Nuc Rbx1:Nuc lamin A/C (G and N) were determined. Data presented are the mean ± standard deviation ratios from three separate experiments. A Student’s *t*-test was used to test for significant differences between U and I conditions. Statistically significant values are indicated by **P < 0.01 and ****P < 0.0001.

**Fig 2:**
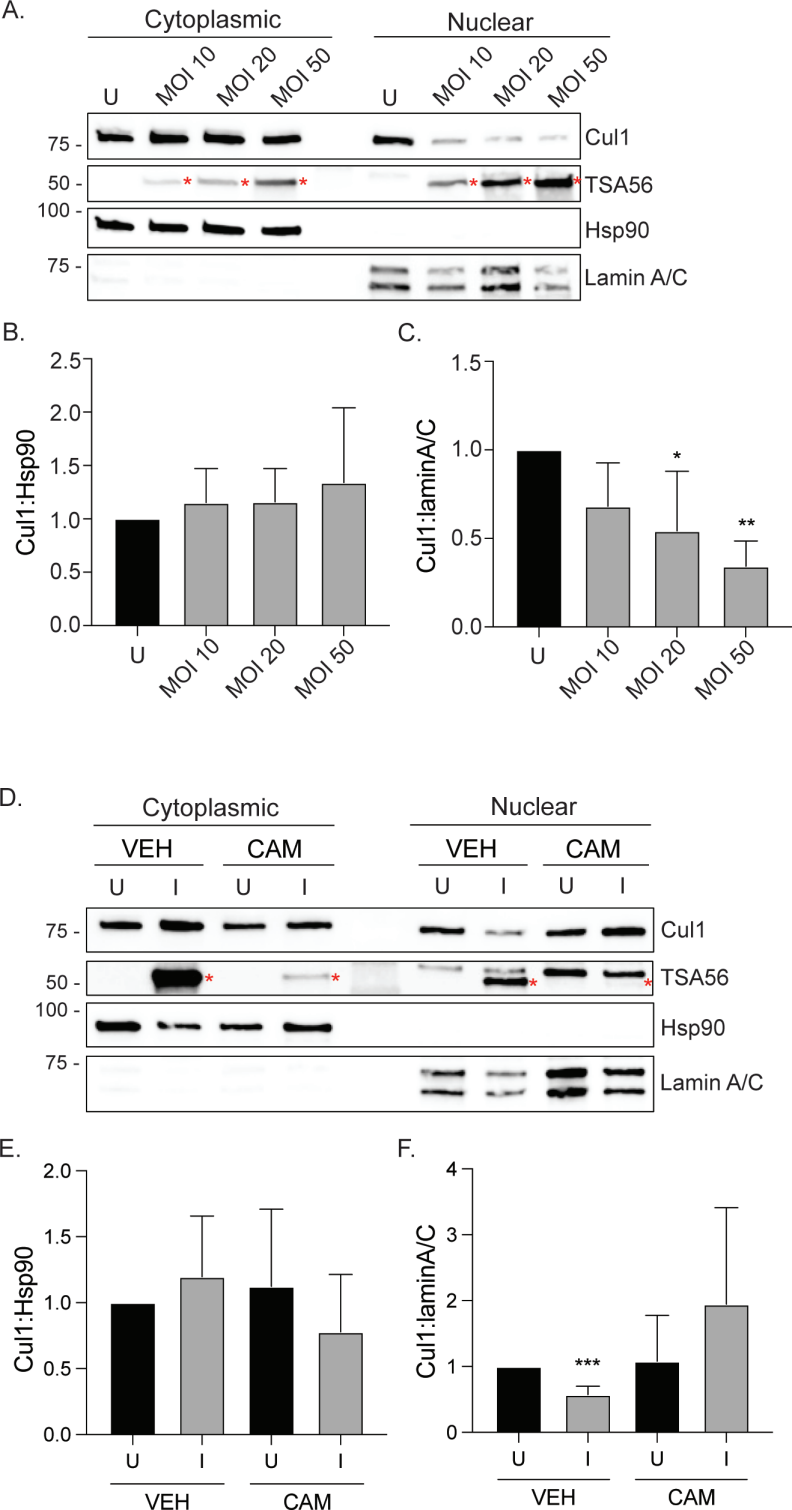
*O. tsutsugamushi* reduces Cul1 nuclear levels in dose- and chloramphenicol-dependent manners. HeLa cells were infected with *O. tsutsugamushi* at an (**A–C**) MOI of 10, 20, or 50 or (**D–F**) treated with chloramphenicol [CAM] or vehicle control [VEH] at 24 hpi. Mock-infected [U] HeLa cells served as a control. Cytoplasmic and nuclear fractions were collected at 24 (**A–C**) or 72 hpi (**D–F**) and subjected to western blot analyses using antibodies specific for Cul1, TSA56, Hsp90, and lamin A/C (**A, D**). Red asterisks denote TSA56 bands. Ratios of the densitometric values normalized to the uninfected control for Cyto Cul1:Cyto Hsp90 (**B, E**) and Nuc Cul1:Nuc lamin A/C (**C, F**) were determined. Data presented are the mean ± standard deviation ratios from three separate experiments. A one-way ANOVA with Dunnett’s *post hoc* test was used to test for significant differences in all conditions compared to the VEH, uninfected control. Statistically significant values are indicated as *P < 0.05, **P < 0.01, and ***P < 0.001.

The SCF ubiquitin ligase is active in the cytoplasm and nucleus (27, 38). Given that *O. tsutsugamushi* commandeers the SCF complex (17, 23, 25, 36), we examined if it alters the subcellular distribution of SCF proteins. HeLa cells were employed, as they are established models for studying *O. tsutsugamushi*–host cell interactions, and their amenability to transfection enables for a correlation between phenotypes observed in infected cells and cells ectopically expressing *Orientia* proteins of interest (17, 18, 20, 23, 25, 36, 40–46). Moreover, the phenomenon that Cul1 regulates c-MYC target gene expression was established using HeLa cells (38). Because *O. tsutsugamushi* infects endothelial cells, RF/6A primate endothelial cells were also included. Infections were performed using *O. tsutsugamushi* Ikeda (hereafter referred to as *O. tsutsugamushi*, unless otherwise necessary), a patient isolate that causes severe disease, and the genome of which is fully annotated (47, 48). Western-blotted nuclear and cytoplasmic fractions of uninfected and *O. tsutsugamushi* infected cells were examined 72 h post infection (hpi) with Cul1, Skp1, and Rbx1 antibodies. Blots were also probed with antibodies specific for the bacterium’s immunodominant outer membrane protein 56 kDa type-specific antigen (TSA56) as an infection control, heat shock protein 90 (Hsp90) or glyceraldehyde-3-phosphate dehydrogenase (GAPDH) as a cytoplasmic fraction loading control, and lamin A/C as a nuclear fraction loading control. Cul1 levels in the nuclear fractions of both cell lines were significantly lower when infected with *O. tsutsugamushi* (Fig. 1). Significant reductions in nuclear Cul1 levels were also observed at 24 hpi in HeLa cells inoculated with different multiplicities of infection (MOI) but not in infected HeLa cells that had been treated with the bacterial translation inhibitor, chloramphenicol (Fig. 2). Thus, *O. tsutsugamushi* promotes reduction of Cul1 levels in the nucleus in bacterial dose- and protein synthesis-dependent manners.

**Fig 3:**
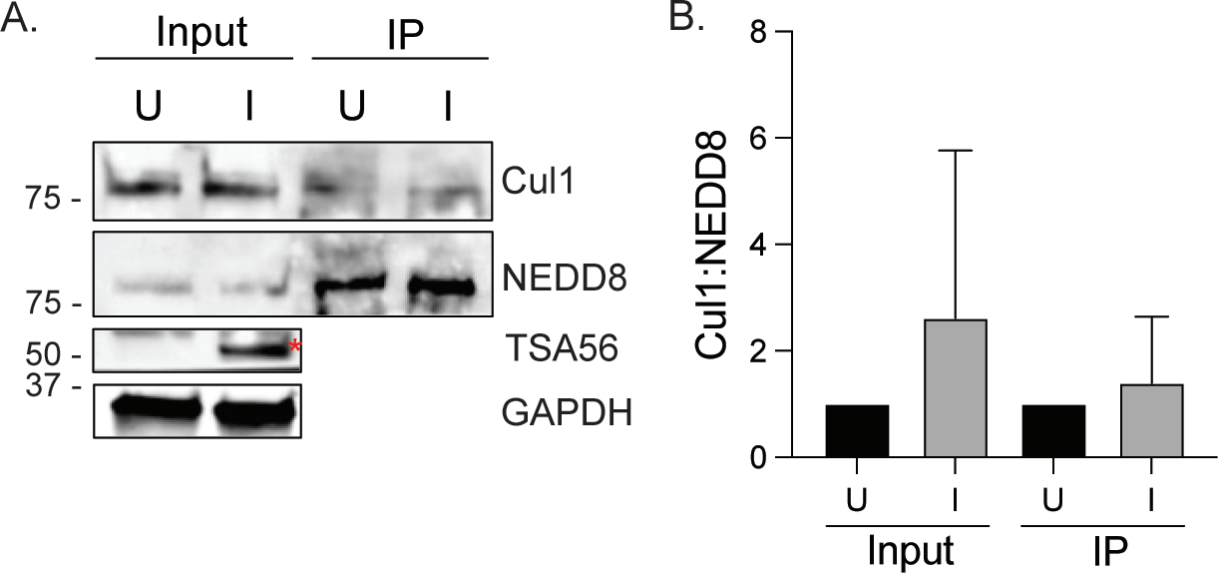
*O. tsutsugamushi* does not impair Cul1 neddylation. Input lysates of mock [U] and *O. tsutsugamushi*-infected [I] HeLa cells were subjected to western blotting with antibody against Cul1 to confirm its presence, TSA56 to affirm infection, and GAPDH to verify that comparable amounts of protein were present per sample (**A**). Red asterisks denote TSA56 bands.Neddylated proteins were immunoprecipitated (IP) from whole-cell lysates. The resulting western blot was probed with Cul1 and NEDD8 antibodies. (**B**) Ratios of the densitometric values for immunoprecipitated Cul1:total immunoprecipitated neddylated protein were determined. Data are representative of four experiments with similar results. Student’s *t*-test determined that there are no significant differences between U and I conditions.

Cullin–RING ligases like the SCF complex are activated by the covalent attachment of the ubiquitin-like molecule, NEDD8 (neural precursor cell expressed, developmentally downregulated 8), to cullins (27). To assess Cul1 neddylation during *O. tsutsugamushi* infection, HeLa cells were infected or not followed by immunoprecipitation of neddylated protein and western blot analysis for Cul1 at 72 h. Cul1 neddylation was not significantly altered (Fig. 3). This result indicates that *O. tsutsugamushi* does not alter Cul1 neddylation and supports that SCF complexes are functional during infection.

### A subset of *O. tsutsugamushi* Anks that bind Cul1 inhibits its nuclear accumulation

**Fig 4:**
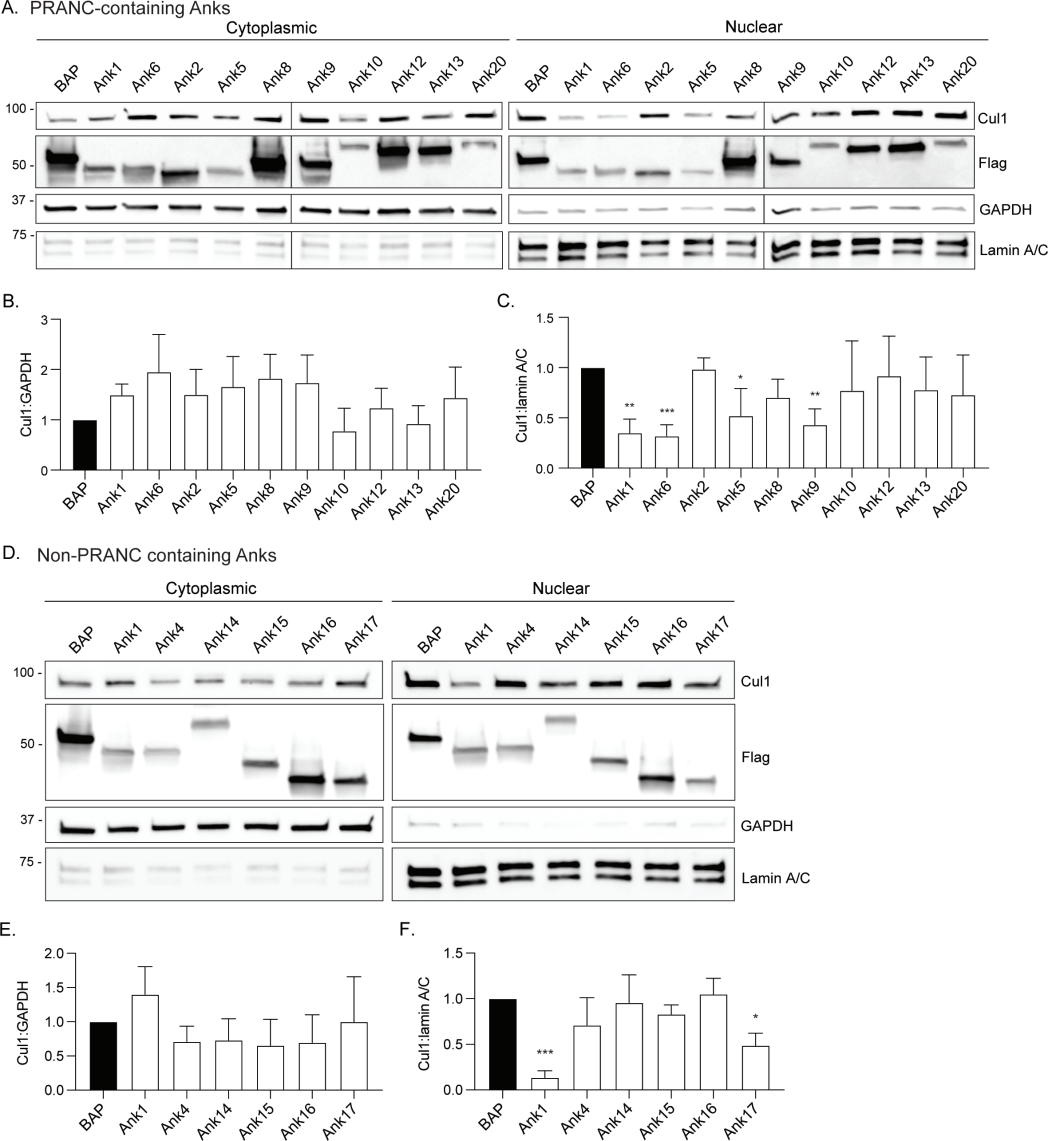
Ank1, Ank5, Ank6, Ank9, and Ank17 inhibit Cul1 nuclear accumulation. HeLa cells were transfected to express Flag-BAP or the indicated Flag-tagged Anks that either carry the PRANC domain (**A**) or do not (**D**). At 16 h, whole cell lysates were separated into cytoplasmic and nuclear fractions that were subjected to western blot analyses using the indicated antibodies. GAPDH and lamin A/C antibodies were used to confirm cytoplasmic and nuclear fraction purities, respectively. Mean ratios ± standard deviation (SD) of Cul1:GAPDH (**B and E**) and Cul1:lamin A/C densitometric signals (**C and F**) from three separate experiments were calculated and normalized relative to the respective ratios for cells expressing Flag-BAP. Data presented are the mean ± SD ratios from the three experiments. A one-way ANOVA with Dunnett’s *post hoc* test was used to test for significant differences compared to Flag-BAP. Statistically significant values are indicated as *P < 0.05, **P < 0.01, and ***P < 0.001.

*O. tsutsugamushi* Ikeda carries 43 Ank ORFs consisting of 17 single-copy and eight multiple identical or near-identical copies totaling 25 distinguishable Anks (35, 47). Eleven of these—Ank1_02 (Ank1), Ank2, Ank5_01 (Ank5), Ank6_02 (Ank6), Ank8, Ank9, Ank10_01 (Ank10), Ank12_01 (Ank12), Ank13, Ank17, and Ank20—carry C-terminal F-box sequences that have been validated to interact with endogenous Cul1, Skp1, and Rbx1 in an F-box-dependent manner. The F-boxes of all of these, except for Ank17, are each part of a PRANC domain. Ank4 has an F-box that weakly binds Skp1 but fails to precipitate Cul1. Ank14, Ank15, and Ank16 have C-terminal sequences that exhibit partial similarity to an F-box but fail to precipitate Cul1 or any other SCF component (25). Like Ank17, the PRANC domain is absent from Ank4, Ank14, Ank15, and Ank16 (23, 25, 47). To assess if any Ank that can bind Cul1 phenocopies the ability of *O. tsutsugamushi* to lower Cul1 levels in the nucleus, HeLa cells were transfected to ectopically express Flag-tagged versions of the 10 PRANC-containing Anks capable of binding Cul1 or Flag-tagged bacterial alkaline phosphatase (BAP) as a negative control. Western blot analyses revealed that Flag-tagged Ank1, Ank5, Ank6, and Ank9 significantly reduced nuclear levels of Cul1 (Fig. 4A through C). When this assay was extended to non-PRANC-containing Anks, only Ank17 was able to significantly reduce Cul1 nuclear accumulation (Fig. 4D through F). The inability of Ank4, Ank14, Ank15, and Ank16 to inhibit Cul1 accumulation in the nucleus is consistent with these proteins being incapable of binding Cul1 (25). Thus, an *O. tsutsugamushi* Ank must carry an F-box that binds Cul1 in order to reduce Cul1 nuclear levels. However, as exhibited by Ank2, Ank8, Ank10, Ank12, Ank13, and Ank20, simply having an F-box that can bind Cul1 does not dictate this ability.

### Ank1 and Ank6 each impede Cul1 nuclear accumulation in an F-box-dependent manner

**Fig 5:**
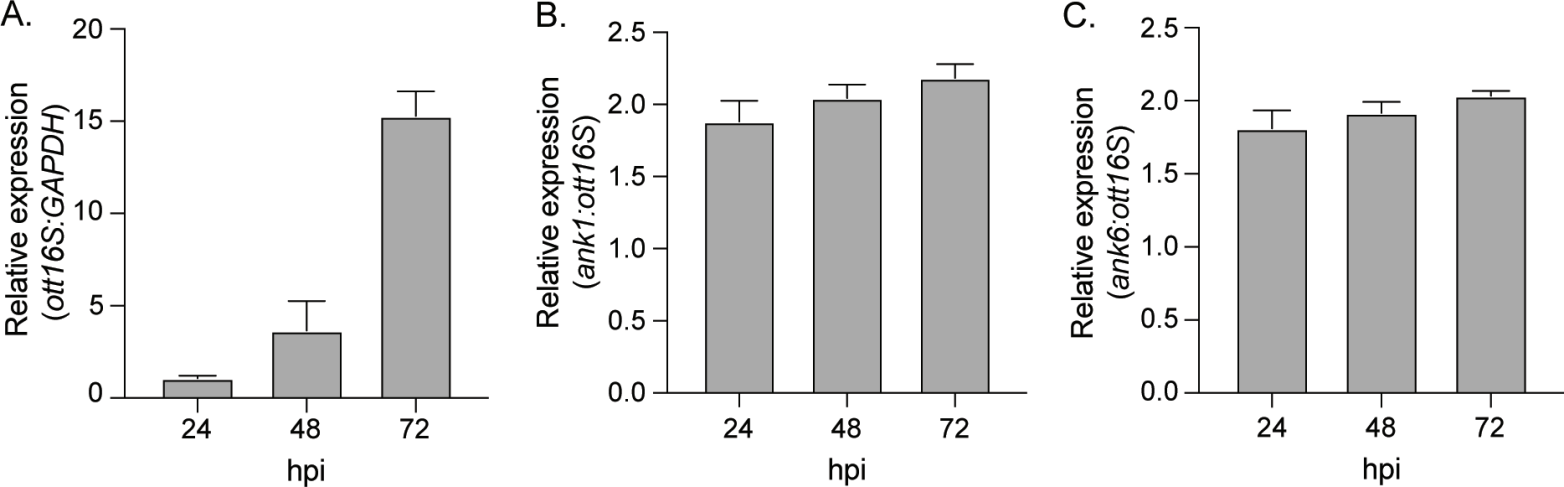
*O. tsutsugamushi* transcriptionally expresses *ank1* and *ank6* throughout infection of host cells. HeLa cells were synchronously infected with *O. tsutsugamushi* followed by collection of total RNA at the indicated time points. RT-qPCR was performed using gene-specific primers. Relative *O. tsutsugamushi* 16S rRNA gene (*ott16S*)-to-human *GAPDH* (**A**), *ank1*-to-*ott16S* (**B**), and *ank6*-to-*ott16S* expression (**C**) was determined using the 2^−ΔΔ^*^CT^* method. Data are representative of three experiments with similar results.

**Fig 6:**
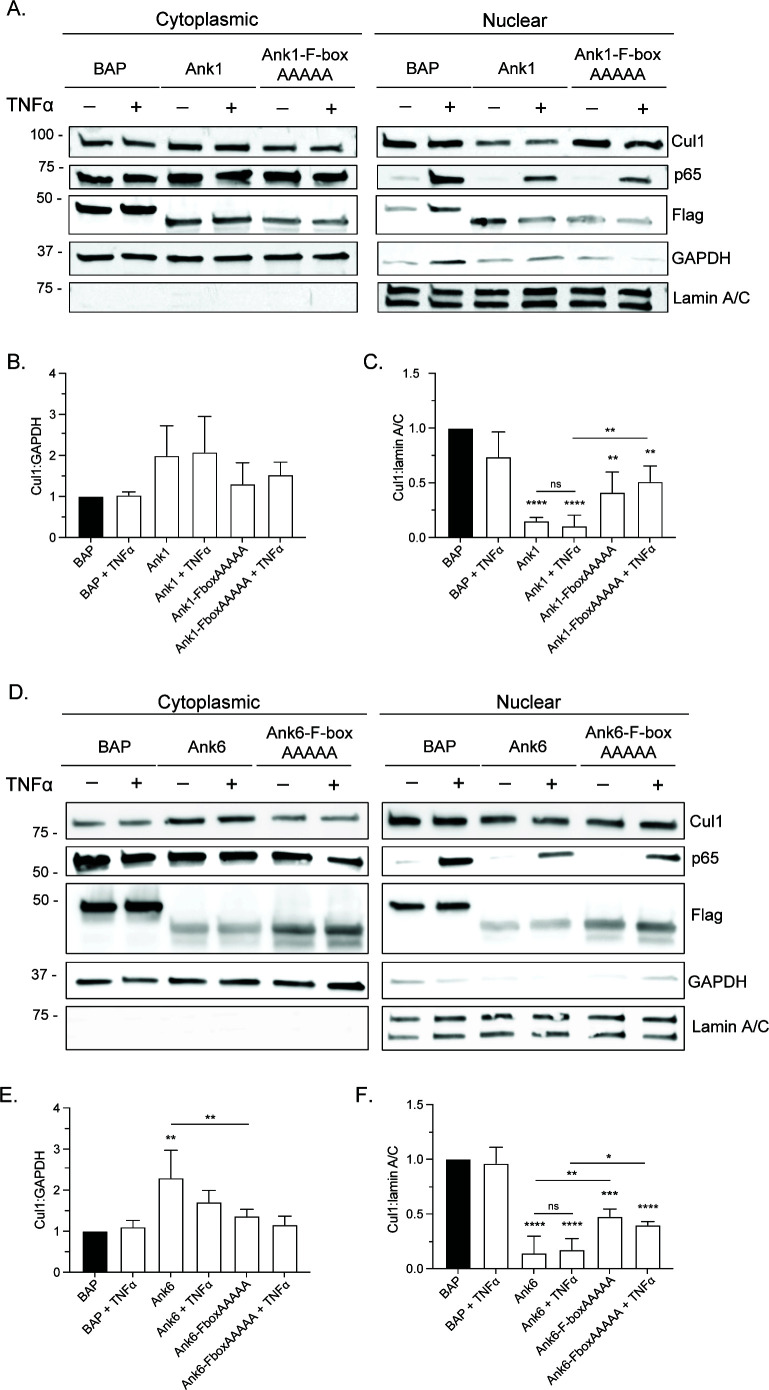
Ank1 and Ank6 inhibition of Cul1 nuclear accumulation critically involves the F-box and occurs independent of TNFα treatment. HeLa cells were transfected to express Flag-tagged BAP, Ank1, Ank1-F-boxAAAAA, Ank6, or Ank6-F-boxAAAAA. At 16 h, the cells were exposed to TNFα or vehicle control for 30 min. (**A and D**) Whole cell lysates were separated into cytoplasmic and nuclear fractions that were subjected to western blot analyses using lamin A/C, GAPDH, p65, Cul1, and Flag epitope antibodies. Mean ratios ± standard deviation of Cul1:GAPDH (**B and E**) and Cul1:lamin A/C densitometric signals (**C and F**) from three separate experiments were calculated and normalized relative to the respective ratios for vehicle-treated cells expressing Flag-BAP. A one-way ANOVA with Dunnett’s or Tukey’s *post hoc* test was used to test for significant differences compared to Flag-BAP or each other, respectively. Statistically significant values are indicated as *P < 0.05, **P < 0.01, ****P < 0.001, and ****P < 0.0001. ns, not significant.

Ank1 and Ank6 were selected as representative Anks to further interrogate the contribution of the F-box to those that reduce Cul1 nuclear levels. These two were chosen because they are among the few Anks for which functional roles have been ascribed. Both inhibit TNFα-stimulated nuclear accumulation of NF-κB p65 in an F-box-dependent manner (25, 36). We first verified that *O. tsutsugamushi* expresses *ank1* and *ank6* throughout infection of HeLa cells (Fig. 5). We could not confirm Ank1 and Ank6 protein expressions because we were unable to raise antisera specific for either that worked sufficiently to be used in western blot or other protein expression analyses. Next, we transfected HeLa cells to express Flag-tagged BAP, Ank1, Ank6, or versions of Ank1 or Ank6 bearing alanine substitutions at positions L1, P2, E4, I9, and D17 of the F-box _1_LPXEXXXXILXXLXXXDLXXX_21_ consensus sequence (F-boxAAAAA). We previously reported that Flag-Ank1-F-boxAAAAA cannot bind Cul1, and that Flag-Ank6-F-boxAAAAA weakly binds Cul1 (25). Because Flag-Ank1 and Flag-Ank6 prevent TNFα-stimulated NF-κB p65 nuclear accumulation in an F-box-dependent manner, cells expressing Flag-tagged Ank1, Ank6, and their F-box mutants were stimulated with TNFα to validate their functionality. NF-κB p65 levels were considerably higher in the nuclear fractions of cells expressing Flag-BAP that had been exposed to TNFα versus vehicle (Fig. 6A through D), thus confirming treatment efficacy. Consistent with previous reports (25, 36), Flag-Ank1 and Flag-Ank6 reduced NF-κB p65 nuclear accumulation in control and TNFα-treated cells (Fig. 6A through D). Cul1 nuclear levels were significantly lower in cells expressing Flag-Ank1 or Flag-Ank6 compared to Flag-Ank1-F-boxAAAAA, Flag-Ank6-F-boxAAAAA, and Flag-BAP (Fig. 6). TNFα had no effect on Cul1 nuclear levels for any condition assessed. These data confirm that a functional F-box capable of interacting with Cul1 is critical for *O. tsutsugamushi* Anks to impede Cul1 accumulation in the nucleus, and that TNFα has no effect on the ability of Ank1 or Ank6 to lower Cul1 nuclear levels.

### Assessment of domain and F-box residue conservation among Anks that modulate Cul1 nuclear levels

**Fig 7:**
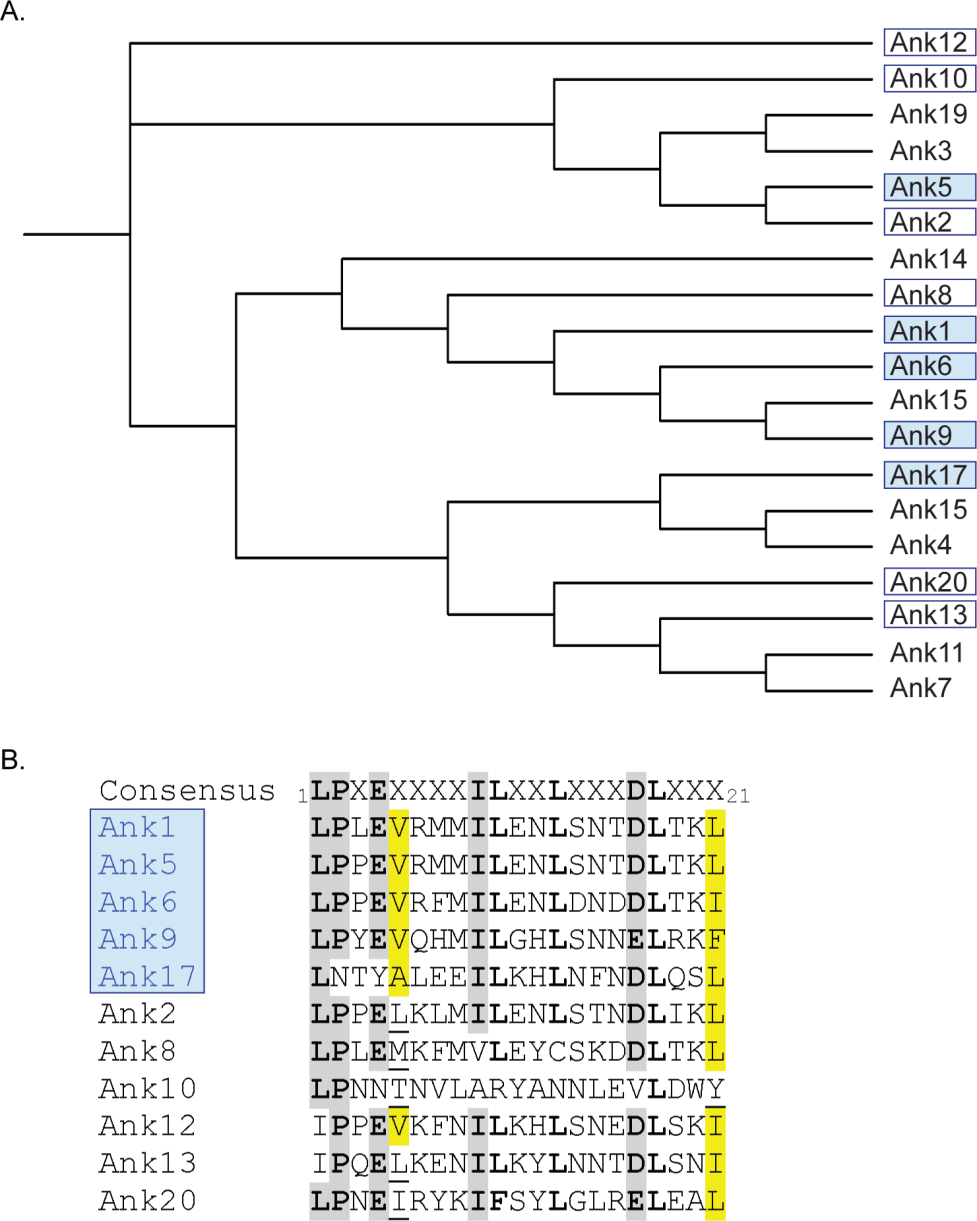
Analyses of *O. tsutsugamushi* Ank AR and F-box domains. (**A**) Phylogenetic tree of the AR domains of the 19 *O. tsutsugamushi* Ikeda anks studied herein. Boxed anks represent those that can bind Cul1. Shaded boxes indicate anks that reduce nuclear Cul1 levels when ectopically expressed, while unshaded boxes denote those that do not. (**B**) Sequence alignment of the 21-amino acid F-box sequence for the 11 Anks that carry an F-box previously validated to bind Cul1. Anks in the blue box reduce Cul1 levels in the nucleus. Highly conserved residues are indicated by boldface text. Gray-highlighted amino acids are those reported to be essential for binding Cul1 and nucleating the SCF complex. Residues that occur at positions 5 and 21 per the F-box consensus in Anks that can lower Cul1 nuclear levels are highlighted in yellow. If residues that occur at these positions in the other Anks are identical or similar to those in Ank1, Ank5, Ank6, Ank9, or Ank17, then they are also highlighted in yellow or underlined, respectively.

Ank1, Ank5, Ank6, Ank9, and Ank17 lower Cul1 nuclear levels in an F-box-dependent manner that involves F-box consensus residue positions 1, 2, 4, 9, and 17. However, the other six Anks that contain F-boxes capable of binding Cul1 fail to alter Cul1 nuclear levels. We rationalized that, perhaps, another Ank domain or additional F-box residues participate in inhibiting Cul1 accumulation in the nucleus. If so, then such domains or residues would be expected to exhibit greater conservation among Anks that modify Cul1 nuclear levels versus those that do not. However, our prior phylogenetic analyses of the F-box and ISR found that both domains in Ank17 diverge considerably from their counterparts in Ank1, Ank5, Ank6, and Ank9 (25). Phylogenetic analysis of the AR domains of the 19 representative Ikeda Anks revealed that those of Ank1, Ank5, Ank6, Ank9, and Ank17 are not more closely related to each other than to those of the other 14 Anks (Fig. 7A). Ank17 lacks a PRANC (23, 25, 47), which rules out this domain’s relevance to modulating Cul1 nuclear levels. Examination of the Ank1, Ank5, Ank6, Ank9, and Ank17 F-boxes for additional conserved residues found that position 5 is a valine (Ank1, Ank5, Ank6, Ank9) or alanine (Ank17), and position 21 is also a hydrophobic residue (leucine in Ank1, Ank5, and Ank17; isoleucine in Ank6; phenylalanine in Ank9) (Fig. 7B). However, these or other hydrophobic residues occur at positions 5 and/or 21 of the six Ank F-boxes that bind Cul1 but do not affect Cul1 nuclear levels. Overall, we could not discern any additional domain or F-box residue that contributes to the abilities of Ank1, Ank5, Ank6, Ank9, and Ank17 to retain the majority of Cul1 in the cytoplasm.

### Cul1 target genes associated with apoptosis are differentially expressed in *O. tsutsugamushi*-infected cells

**Fig 8:**
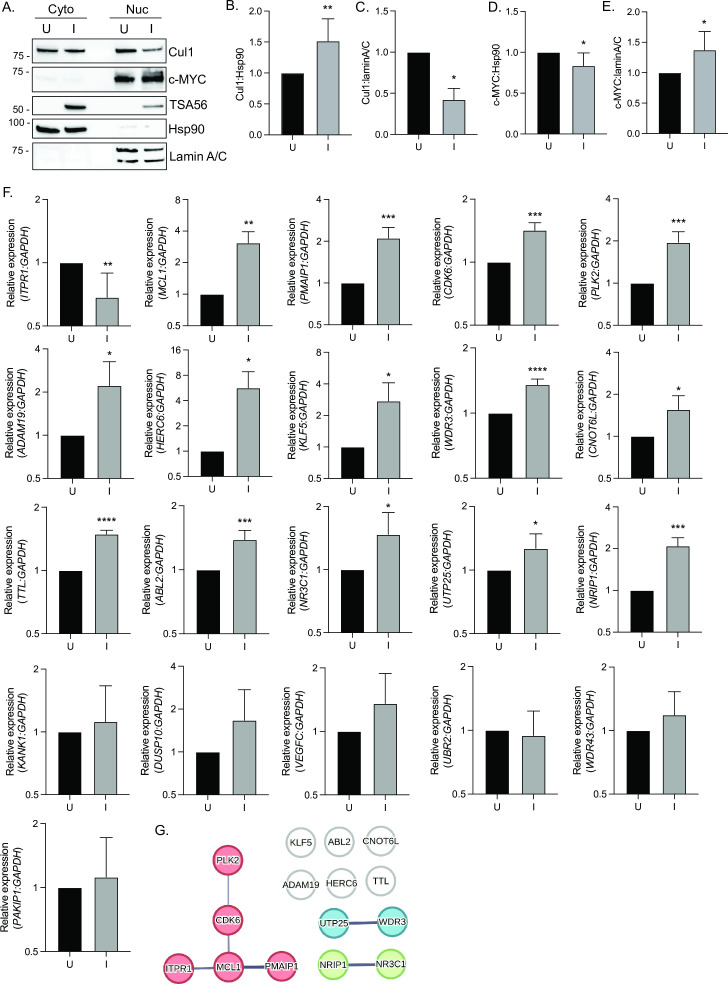
*O. tsutsugamushi*-infected cells have elevated c-MYC nuclear levels and exhibit increased transcription of Cul1-regulated genes, including several involved in apoptosis. HeLa cells were infected with *O. tsutsugamushi* [I] or mock-infected [U]. At 72 hpi, cytoplasmic [Cyto] and nuclear [Nuc] fractions were subjected to western blot analyses using the indicated antibodies (**A**). Hsp90 and lamin A/C antibodies were used to confirm cytoplasmic and nuclear fraction purities, respectively. Ratios of the densitometric values for Cyto Cul1:Cyto Hsp90 (**B**), Nuc Cul1:Nuc lamin A/C (**C**), Cyto c-MYC:Cyto Hsp90 (**D**), and Nuc c-MYC:Nuc lamin A/C (**E**) were determined. (**F**) RT-qPCR was performed on RNA from U or I HeLa cells collected at 72 hpi. Relative expression ± standard deviation (SD) of the indicated genes normalized to that of *GAPDH* was determined using the 2^−ΔΔ^*^CT^* method. (**G**) STRING analysis of differentially expressed Cul1-regulated genes. STRING analysis with Markov clustering was employed to define prominent clusters of the 15 genes that exhibit significantly different expressions between U and I cells (**F**). Solid lines denote a direct connection between genes, while line thickness indicates the strength of the connection. The red clustered nodes are in the BCL-2 family protein complex GO biological process. Blue and green clustered nodes do not associate with any GO biological process term. White nodes do not cluster with other genes. Data presented are the mean ± SD ratios from three to four independent experiments. Statistical significance was evaluated by Student’s *t*-test between U and I conditions. Statistically significant values are indicated as *P < 0.05, **P < 0.01, **P < 0.001, and ****P < 0.0001.

Cul1 was recently discovered to act as a transcriptional repressor that preferentially associates with chromatin at active promoter sequences and sites of proteasome degradation-prone ubiquitination. Cul1 is postulated to antagonize c-MYC based on observations that more than 67% of Cul1 target genes is controlled by c-MYC; Cul1 depletion increases c-MYC-regulated gene expression; and Cul1 overexpression reduces expression of these genes (38). To assess changes in Cul1 and c-MYC levels in the nucleus during *O. tsutsugamushi* infection, we compared the relative abundances of both proteins in nuclear fractions from infected and uninfected cells. *O. tsutsugamushi*-infected cells exhibited significantly decreased levels of Cul1 in the nuclear fraction, while c-MYC nuclear levels were significantly increased (Fig. 8A through E). Given these results, we hypothesized that *O. tsutsugamushi* alters the expression of a subset of genes that can be regulated by the interplay between Cul1 and c-MYC. Cross-referencing a published list of 2,704 Cul1 target genes (38) with our RNAseq analysis of *O. tsutsugamushi*-infected HeLa cells (17) identified 107 Cul1 target genes that are upregulated during infection (Data Set S1). Upon re-examining the gene ontology (GO) biological process terms assigned to genes upregulated at 48 hpi (17), we found that 358 GO terms each contained 1 to 10 of the 107 Cul1 target genes. Focusing on the biological processes into which at least six genes were categorized revealed that *O. tsutsugamushi* infection upregulates Cul1-regulated genes that are typical of cellular responses to an intracellular microbe: response to bacterium, response to virus, defense response to other organisms, defense response to virus, response to molecule of bacterial origin, and response to lipopolysaccharide (Table 1). Several of the remaining GO terms could be generally categorized into processes that c-MYC transcriptionally regulates and that *O. tsutsugamushi* potentially modulates to its advantage, including cell proliferation, signal transduction, and apoptosis (Tables 1 and 2) (49). Twenty-one representative Cul1 target genes from these categories were selected for RT-qPCR analysis at 72 hpi when Cul1 levels are decreased, and c-MYC levels are increased in the nucleus (Fig. 8A through E). Fourteen were significantly upregulated, and one was significantly downregulated in infected cells (Fig. 8F). The Search Tool for the Retrieval of Interacting Genes/Proteins (STRING) assessment with Markov clustering (50) of the 15 differentially expressed genes found that five functionally cluster in the B-cell lymphoma 2 (BCL-2) family (Fig. 8G). Specifically, genes encoding anti-apoptotic factors cyclin-dependent kinase 6 (CDK6), myeloid cell leukemia-1 (MCL1), Polo-like kinase-2 (PLK2), and proapoptotic phorbol-12-myristate-13-acetate (PMA)-induced protein 1 (PMAIP1) are upregulated. Another proapoptotic factor, inositol 1,4,5 triphosphate receptor-1 (ITPR1) is downregulated (Fig. 8F and G). Collectively, the *O. tsutsugamushi*-induced decrease in levels of Cul1 in the nucleus (38), differential expression of BCL2 family genes that are regulated by Cul1, and increase in c-MYC nuclear levels suggest the promotion of an anti-apoptotic phenotype.

**TABLE 1:** Cul1 target genes that are upregulated in *O. tsutsugamushi*-infected HeLa cells^*a*^

GO ID^*b*^	GO biological process	Gene products
GO:0009617	Response to bacterium	CD55, **DUSP10^*c*^**, **HERC6**, HMCN1, JUNB, PTGS2, PTGER4, SHC1, SLC17A5, **WDR3**
GO:0022613	Ribonucleoprotein complex biogenesis	BICD1, **CNOT6L**, **UTP25**, GTPBP10, **PAK1IP1**, PRKAA2, **TTL**, URB2, **WDR3**, **WDR43**
GO:0071407	Cellular response to organic cyclic compound	DDIT4, DDX18, DDX58, GNG2, IFIH1, **KLF5**, **NR3C1**, **NRIP1**, PDE12, PTGS2
GO:1902532	Negative regulation of intracellular signal transduction	**ABL2**, CNKSR3, DDIT4, **DUSP10**, DYRK2, **ITPR1**, **KANK1**, **MCL1**, PRKAA2, PTGS2
GO:0018212	Peptidyl–tyrosine modification	**ABL2**, DYRK2, FGFR1, IBTK, RAP2C, ROR1, SHC1, TMEM102, TTL
GO:0009615	Response to virus	**CDK6**, DDIT4, DDX58, IFIH1, IFIT2, IFIT5, PDE12, **PMAIP1**
GO:0018108	Peptidyl–tyrosine phosphorylation	**ABL2**, DYRK2, FGFR1, IBTK, PDE12, ROR1, SHC1, TMEM102
GO:0062012	Regulation of small-molecule metabolic process	DDIT4, DYRK2, PDE12, PFKFB2, **PMAIP1**, PRKAA2, PTGS2, SLC17A5
GO:0072507	Divalent inorganic cation homeostasis	**ABL2**, CD55, IBTK, **ITPR1**, PTGER4, SLC30A7, SLC39A10, THADA
GO:0098542	Defense response to other organisms	DDIT4, DDX58, IFIH1, IFIT2, IFIT5, **PMAIP1**, PDE12, SHC1
GO:0001525	Angiogenesis	FGFR1, **KLF5**, NRXN3, **PLK2**, PTGS2, SHC1, **VEGFC**
GO:0010975	Regulation of neuron projection development	**ABL2**, FGFR1, FZD1, **KANK1**, PDLIM5, **PLK2**, **TTL**
GO:0042060	Wound healing	C4BPB, **DUSP10**, GNG2, **ITPR1**, **KANK1**, **KLF5**, SLC7A11
GO:0042254	Ribosome biogenesis	UTP25, GTPBP10, **PAK1IP1**, **TTL**, **URB2**, **WDR3**, **WDR43**
GO:0051607	Defense response to virus	DDIT4, DDX58, IFIH1, IFIT2, IFIT5, PDE12, **PMAIP1**
GO:0001503	Ossification	**CDK6**, EXT1, FZD1, JUNB, PTGS2, PTGER4
GO:0002237	Response to molecule of bacterial origin	CD55, **DUSP10**, JUNB, PTGER4, PTGS2, **WDR3**
GO:0002790	Peptide secretion	DDX58, IFIH1, **ITPR1**, PFKFB2, PTGER4, **VEGFC**
GO:0009306	Protein secretion	DDX58, IFIH1, **ITPR1**, PFKFB2, PTGER4, **VEGFC**
GO:0010631	Epithelial cell migration	**DUSP10**, FGFR1, **KANK1**, **PLK2**, PTGER4, **VEGFC**
GO:0016055	Wnt signaling pathway	FZD1, GNG2, **KANK1**, PRKAA2, ROR1, TLE4
GO:0032496	Response to lipopolysaccharide	CD55, DUSP10, JUNB, PTGER4, PTGS2, **WDR3**
GO:0036293	Response to decreased oxygen levels	DDIT4, **ITPR1**, NAMPT, PTGS2, **PMAIP1**, **VEGFC**
GO:0045785	Positive regulation of cell adhesion	**ADAM19**, CD55, **CDK6**, **DUSP10**, IL7R, TMEM102
GO:0050708	Regulation of protein secretion	DDX58, IFIH1, **ITPR1**, PFKFB2, PTGER4, VEGFC
GO:0050900	Leukocyte migration	EPS8, PTGER4, SHC1, SLC7A11, TMEM102, **VEGFC**
GO:0070482	Response to oxygen levels	DDIT4, **ITPR1**, NAMPT, **PMAIP1**, PTGS2, **VEGFC**
GO:0090130	Tissue migration	**DUSP10**, FGFR1, **KANK1**, **VEGFC**, **PLK2**, PTGER4
GO:0090132	Epithelium migration	**DUSP10**, FGFR1, **KANK1**, **VEGFC**, **PLK2**, PTGER4
GO:0198738	Cell–cell signaling by wnt	FZD1, GNG2, **KANK1**, PRKAA2, ROR1, TLE4
GO:1990823	Response to leukemia inhibitory factor	EPS8, **KLF5**, GNPNAT1, TLE4, VEGFC, WDR3
GO:1990830	Cellular response to leukemia inhibitory factor	EPS8, **KLF5**, GNPNAT1, TLE4, VEGFC, WDR3
GO:1901652	Response to peptide	**KANK1**, **KLF5**, NAMPT, PTGS2, SHC1, TBC1D4

^*a*^ Cul1-target genes (37) that are upregulated in *O. tsutsugamushi*-infected HeLa cells (16). Included are the GO biological processes into which at least six genes were categorized. The entire list is provided in Data Set S1.

^*b*^ ID, identification.

^*c*^ Genes indicated by boldface text are those that were selected for RT-qPCR validation studies.

**TABLE 2:** Cul1 target genes selected for RT-qPCR analyses

Biological group	Gene	Gene product function
Proliferation	ADAM19	Metalloproteinase linked to cell migration, cell adhesion, cell–cell and cell–matrix interactions, and signal transduction
	CDK6	Catalytic subunit of the protein kinase complex that is important for cell cycle G1 phase progression and G1/S transition
	KLF5	Kruppel-like factor subfamily of zinc finger protein and transcriptional activator that may participate in both promoting and suppressing cell proliferation
	WDR3	Proteins belonging to the WD repeat family are involved in a variety of cellular processes, including cell cycle progression, signal transduction, apoptosis, and gene regulation.
	KANK1	Involved in the control of cytoskeleton formation by regulating actin polymerization, establishment, and persistence of cell polarity during directed cell movement in wound healing. In the nucleus, it is involved in beta-catenin-dependent activation of transcription.
	DUSP10	Dual-specificity protein phosphatases inactivate their target kinases by dephosphorylating phosphoserine/threonine and phosphotyrosine residues to negatively regulate members of the MAP kinase superfamily, which is associated with cellular proliferation and differentiation.
	CNOT6L	Predicted to enable poly(A)-specific ribonuclease activity and involved in positive regulation of cell population proliferation and positive regulation of cytoplasmic mRNA processing body assembly
	TTL	Cytosolic enzyme that restores the tyrosine residues after microtubule disassembly
	PLK2	Tumor suppressor serine/threonine–protein kinase involved in synaptic plasticity, centriole duplication, and G1/S phase transition
	VEGFC	Promotes angiogenesis and endothelial cell growth and can also affect the permeability of blood vessels
	NR3C1	Glucocorticoid receptor that functions as both a transcription factor that binds to glucocorticoid response elements in the promoters of glucocorticoid responsive genes to activate their transcription and as a regulator of other transcription factors; it is involved in inflammatory responses, cellular proliferation, and differentiation in target tissues
Ribosome biogenesis, RNA binding	URB2	Predicted to be involved in ribosome biogenesis; essential for hematopoietic stem cell development through the regulation of p53/TP53 pathway
	WDR43	Enables RNA binding activity; involved in positive regulation of rRNA processing and positive regulation of transcription by RNA polymerase I
	UTP25	Enables RNA binding activity; involved in protein catabolic processes, protein destabilization, protein localization to nucleolus, and embryonic development through regulation of the p53 pathway
Signal transduction	ITPR1	Encodes an intracellular receptor for inositol 1,4,5-trisphosphate that mediates calcium release from the endoplasmic reticulum
	PAK1IP1	Negatively regulates the PAK1 kinase
Circadian rhythm	NRIP1	Modulates transcriptional activation by steroid receptors, such as NR3C1, NR3C2, and ESR1
Apoptosis	MCL1	Involved in the regulation of apoptosis versus cell survival and in the maintenance of viability but not of proliferation
	PMAIP1	Promotes activation of caspases and apoptosis; promotes mitochondrial membrane changes and efflux of apoptogenic proteins from the mitochondria; contributes to p53/TP53-dependent apoptosis; promotes proteasomal degradation of MCL1; competes with BAK1 for binding to MCL1 and can displace BAK1 from its binding site on MCL1; and competes with BIM/BCL2L11 for binding to MCL1 and can displace BIM/BCL2L11 from its binding site on MCL1
Miscellaneous	HERC6	Ubiquitin ligase predicted to catalyze the formation of a thioester with ubiquitin before transferring it to a substrate
	ABL2	Member of the Abelson family of nonreceptor tyrosine protein kinases; plays a role in cytoskeletal rearrangements through its C-terminal F-actin- and microtubule-binding sequences

### *O. tsutsugamushi* inhibits apoptosis in HeLa cells

**Fig 9:**
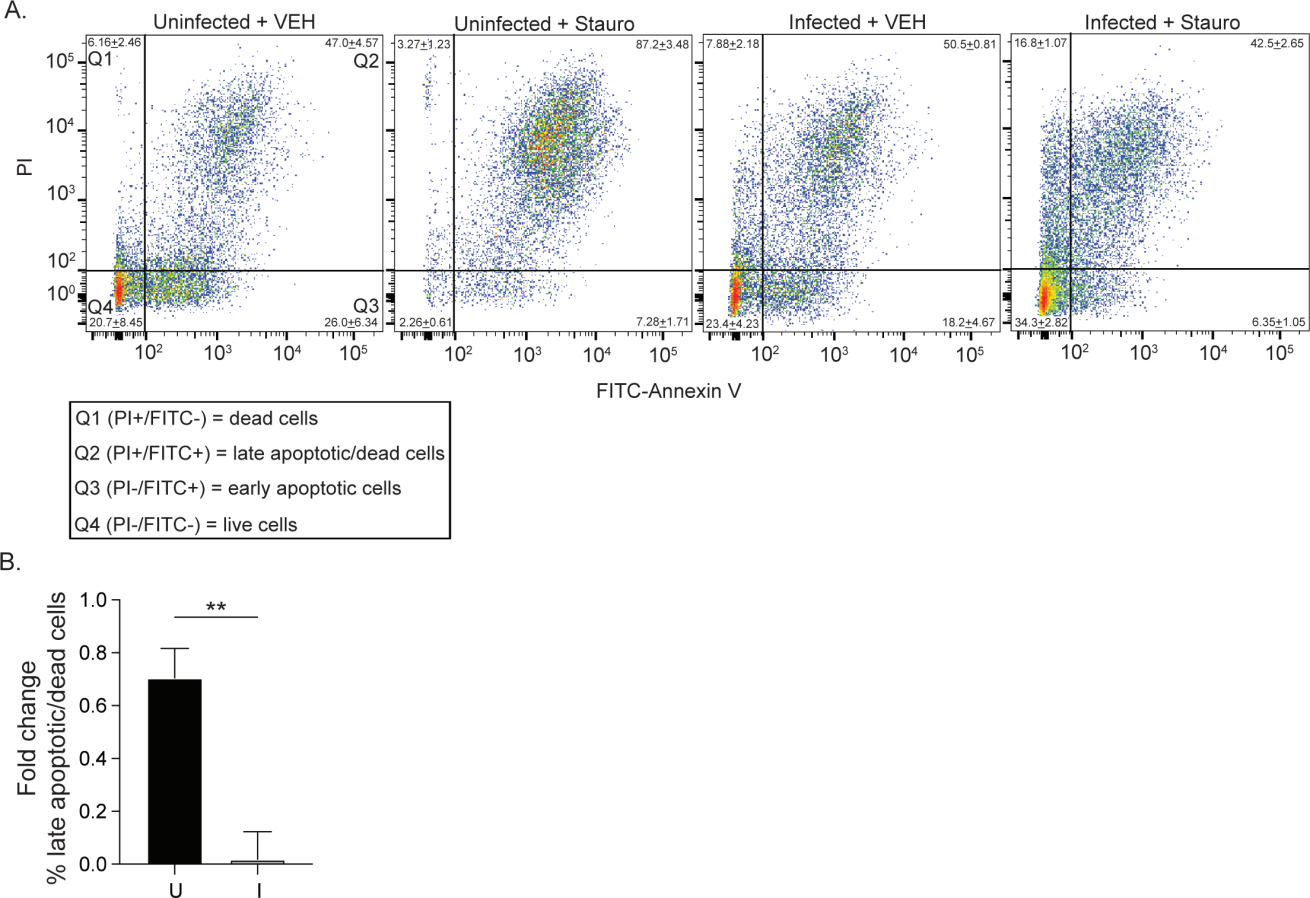
*O. tsutsugamushi*-infected cells resist apoptosis. HeLa cells were infected with *O. tsutsugamushi* for 48 h [I] before treatment with staurosporine [Stauro] or vehicle [VEH]. Mock infected [U] served as an uninfected control. After 24 h, U and I samples were incubated with FITC–annexin V and PI and analyzed by flow cytometry. (**A**) The quadrant [Q] layout for the dot plot of uninfected + VEH is the same for all samples. Q1 [PI+/FITC−] and Q2 [PI+/FITC+] indicate late apoptotic and/or dead cells. Q3 [PI−/FITC+] shows early apoptotic cells. Q4 [PI−/FITC−] has live cells. The mean ± standard deviation (SD) percentages of cells per quadrant for three independent experiments is indicated. (**B**) The fold change in the mean percentage ± SD of late apoptotic/dead cells [Q1–2] from Stauro- compared to VEH-treated counterparts from three separate experiments was determined. Statistical significance was evaluated by Student’s *t*-test between U and I conditions. Statistically significant values are indicated as **P < 0.01.

*O. tsutsugamushi* delays apoptosis in endothelial and fibroblast cell lines until late in infection when a high bacterial burden has been reached (51, 52). The bacterium also inhibits pharmacologic induction of intrinsic apoptosis in monocytic cells (53). Because *Orientia* elevates nuclear levels of c-MYC and alters expression of apoptosis genes in HeLa cells, we investigated if it similarly impairs apoptosis in this cell line. *O. tsutsugamushi* and uninfected control cells were treated with staurosporine, an inducer of the intrinsic apoptosis pathway, or vehicle at 48 hpi. Fluorescein isothiocyanate (FITC)-conjugated annexin V and propidium iodide (PI) were added, followed by flow cytometric analysis. Annexin V binds surface-exposed phosphatidylserine in apoptotic cells. PI is a membrane impermeant dye that stains DNA when the membrane becomes permeable during host cell death. The combined percentage of late apoptotic (annexin V- and PI-positive) and dead cells (PI-positive) was significantly higher in uninfected cells compared to infected cells following staurosporine treatment. When comparing the percentage of late apoptotic/dead cells in staurosporine-treated samples to that of vehicle-treated controls, infection robustly inhibited staurosporine-induced apoptosis (Fig. 9). Thus, *O. tsutsugamushi* inhibits intrinsic apoptosis in HeLa cells.

### Apoptosis resistance conferred by ectopically expressed *O. tsutsugamushi* Anks is dependent on the ability to inhibit Cul1 nuclear accumulation

**Fig 10:**
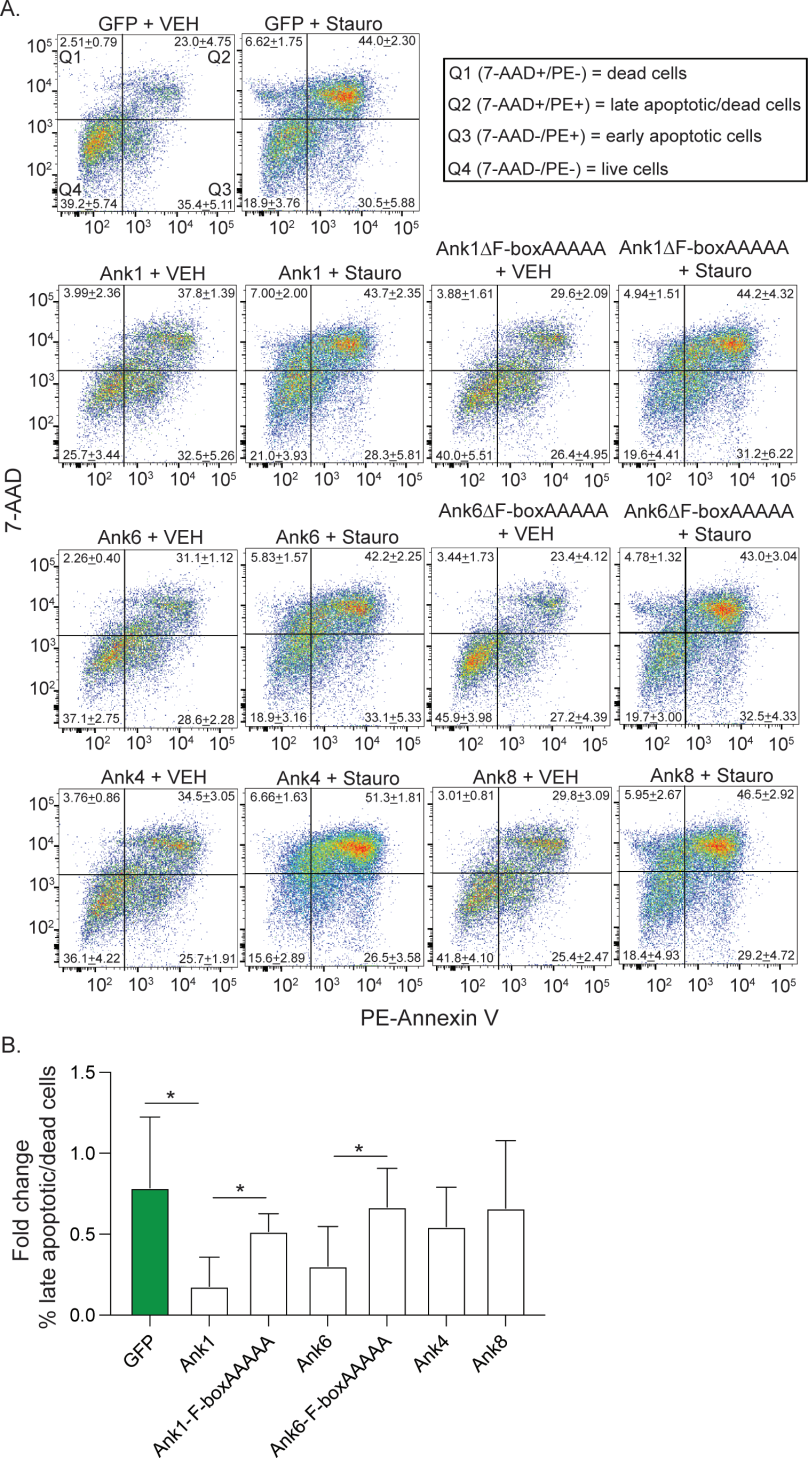
Ectopically expressed Ank1 and Ank6 inhibit apoptosis in an F-box-dependent manner. HeLa cells were transfected to express GFP-Ank1, -Ank1-F-boxAAAAA, -Ank6, -Ank6-F-boxAAAAA, -Ank4, -Ank8, or GFP alone for 18 h. The cells were then treated with staurosporine [Stauro] or vehicle [VEH] for 24 h, incubated with PE–annexin V and 7-AAD, and analyzed by flow cytometry. (**A**) The quadrant [Q] layout for the dot plot of uninfected + VEH is the same for all samples. Q1 [7-AAD+/PE−] and Q2 [7-AAD+/PE+] indicate late apoptotic and/or dead cells. Q3 [7-AAD−/PE+] shows early apoptotic cells. Q4 [7-AAD−/PE−] has live cells. The mean ± standard deviation (SD) percentages of cells per quadrant for three independent experiments were indicated. (**B**) The fold change in the mean percentage ± SD of late apoptotic/dead cells [Q1–2] from Stauro- compared to VEH-treated counterparts from three separate experiments was determined. Statistical significance was evaluated by one-way ANOVA, followed by Tukey’s *post hoc* test for comparison of each sample to each other or each sample to the VEH-treated GFP control. Statistically significant values are indicated as *P < 0.05.

Cul1 levels are significantly decreased, and c-MYC nuclear levels are significantly increased in nuclear fractions of *O. tsutsugamushi*-infected cells, and these phenomena correlate with an impairment of apoptosis. Furthermore, a subset of the *Orientia* Anks (Ank1, Ank5, Ank6, Ank9, Ank17) that bind Cul1 in an F-box-dependent manner impede Cul1 nuclear localization. To determine if representative Anks from this subset also inhibit apoptosis, HeLa cells were transfected to express GFP-tagged Ank1, Ank6, F-boxAAAAA versions thereof, or GFP alone. Cells expressing GFP-Ank4, which neither binds nor excludes Cul1 from the nucleus, and Ank8, which binds but cannot inhibit Cul1 nuclear accumulation [Fig. 4 and (25)], were included to further differentiate the contribution of Cul1 binding versus Cul1 nuclear exclusion to apoptosis inhibition. 7-Aminoactinomycin D (7-AAD) is another membrane impermeant dye that stains DNA upon induction of apoptosis. The amount of staurosporine-induced death in cells expressing GFP-Anks or GFP was defined as the percentage of late apoptotic (annexin V- and 7-AAD-positive) and dead cells (7-AAD-positive). Only cells expressing GFP-Ank1 and GFP-Ank6 resisted staurosporine-induced apoptosis (Fig. 10). This result establishes that Ank inhibition of intrinsic apoptosis requires F-box-dependent exclusion of Cul1 from the nucleus and not merely binding Cul1.

## DISCUSSION

As an obligate intracellular microbe, *O. tsutsugamushi* must counter innate defenses to gain an advantage in its tug-of-war with eukaryotic host cells to maximize its survival. Indeed, although apoptosis contributes to the anti-*Orientia* response *in vivo* (54, 55), multiple *in vitro* studies established that the pathogen delays programmed death of infected cells (51–53). Because *O. tsutsugamushi* does not initiate exponential growth until between 12 and 24 h and does not begin to exit cells until 72 to 96 h (22, 56, 57), this strategy presumably gives the microbe sufficient time to replicate and initiate spread to naïve cells before losing its intracellular niche to cell death. c-MYC can exert pro- or anti-apoptotic transcriptional regulation depending on the cellular landscape. In the context of BCL-2 family gene upregulation, c-MYC induces an anti-apoptotic phenotype (49, 58, 59).

We found that *O. tsutsugamushi* modulates the balance between Cul1 and c-MYC in the nucleus, resulting in an overall BCL-2 anti-apoptotic gene expression profile and resistance to staurosporine-induced apoptosis. Cul1 and c-MYC bind chromatin at the E-box and simultaneously do so at sites of ubiquitination and proteasome activity, which has led to the prevailing model that Cul1 spatially and selectively directs c-MYC degradation at promoter sites (38). Thus, by excluding Cul1 from the nucleus, *Orientia* stabilizes nuclear levels of c-MYC, thereby promoting its ability to regulate gene expression. Cul1 nuclear exclusion is not due to a difference in its neddylation status, as this occurs comparably in uninfected and infected cells. *Orientia* prevents nuclear accumulation of Cul1 in a dose-dependent manner and requires bacterial protein synthesis to do so, indicating the involvement of one or more oriential virulence factors. The recalcitrance to apoptosis exhibited by infected cells is phenocopied by uninfected cells expressing Ank1 or Ank6, two representatives of the five Anks that block Cul1 nuclear accumulation in an F-box-dependent manner. As exemplified by ectopically expressed Ank8, the remaining six F-box-containing Anks that bind but do not alter Cul1 levels in the nucleus likely do not hinder Cul1-mediated apoptosis. Therefore, a subset of these effectors exhibits specificity for modulating Cul1 nuclear levels that extend beyond simply having an F-box. Interestingly, *O. tsutsugamushi* infection partially mimics the activity of host FBXL16 (F-box and leucine-rich repeat protein 16), which antagonizes Skp1 but not Cul1 of the SCF E3 ubiquitin ligase that ubiquitinates c-MYC to regulate its cellular levels via proteasomal degradation (60). While FBXL16 is an oncoprotein that promotes cancer cell growth and migration by increasing c-MYC stability, *Orientia* evolved as an obligate intracellular microbe to stabilize c-MYC and extend the life of its host cell.

Of the 11 Anks that bind Cul1 in an F-box-dependent manner, only Ank1, Ank5, Ank6, Ank9, and Ank17 prevent Cul1 from accumulating in the nucleus. Ectopically expressed Ank1 and Ank6 require the F-box to accomplish this task; however, a comparison of all *O. tsutsugamushi* Ank F-box sequences found no variation in their amino acid residues that could account for this differential ability. These five Anks might selectively exclude Cul1 from the nucleus through the cooperative actions of the F-box and another domain. Prior evidence suggests unlikely involvement of the ISR or PRANC (23, 25, 47). While the AR domains of Ank1, Ank5, Ank6, Ank9, and Ank17 do not phylogenetically cluster, we cannot exclude the possibility that one or more of these Anks’ ARs bind Cul1 or influence its subcellular localization. Precedent for the first scenario is provided by *O. tsutsugamushi* Ank5, which sequesters NLRC5 in the cytoplasm using its fourth AR (22).

*O. tsutsugamushi* alters mitochondrial morphology and impairs mitochondrial fitness (57, 61). It could combat mitochondrial stress-induced cell death through the actions of the three Cul1-regulated BCL-2 family genes that are differentially expressed during infection and synergize with c-MYC to inhibit apoptosis (49, 58, 59). MCL1, which is upregulated, inhibits mitochondrial cytochrome c release (62). PLK2, also upregulated, mediates cell survival when mitochondrial respiration is impaired (63). ITPR1, which is downregulated, encodes an intracellular channel that localizes to endoplasmic reticulum (ER)–mitochondria contact sites and facilitates calcium release from the ER into the mitochondria (64, 65). Prior to this study, the only known *O. tsutsugamushi* anti-apoptotic mechanism was inhibition of ER calcium release (53). Perhaps, ITPR1 downregulation is responsible. PMAIP1, which is upregulated, is a proapoptotic member of the BCL-2 family that has alternative functions in autophagy and metabolism that could, in tandem with other host signals, play a beneficial role during *O. tsutsugamushi* infection (66, 67). The other BCL-2 family gene upregulated during infection is CDK6, which facilitates cell cycle progression and is associated with c-MYC-induced cell proliferation (68) and hence could counteract apoptosis.

Like all obligate intracellular bacteria, *O. tsutsugamushi* must maximally use the limited number of proteins that its reduced genome encodes. In this regard, our study reinforces the Anks’ multifunctional nature for manipulating host immune defenses. In addition to these effectors using their AR and F-box domains to direct ubiquitination and proteasomal degradation of NLRC5, as exemplified by Ank5 (22), or to modulate NF-κB function, as epitomized by Ank1 and Ank6 (25, 36), multiple *Orientia* Anks use the F-box to exclude Cul1 from the nucleus and impair apoptosis. *O. tsutsugamushi* Ikeda Ank1, Ank5, and Ank6 are encoded by multicopy genes, while Ank9 and Ank17 are single-copy (21, 35, 47). Hence, Ikeda can express a combined total of 11 Anks capable of altering the Cul1:c-MYC ratio in the nucleus. Of the seven other annotated *O. tsutsugamushi* strain genomes, at least one copy of Ank1 is found in two (Kato, UT76), Ank5 in one (UT76), Ank6 in five (Boryong, Karp, TA686, UT76, UT176), Ank9 in six (Gilliam, Karp, Kato, TA686, UT76, UT176), and Ank17 in three (Gilliam, Karp, UT176) (35), affirming that the ability to modulate Cul1 nuclear levels is likely conserved among strains.

Overall, we report that *O. tsutsugamushi* dysregulates Cul1 levels in the nucleus, which elevates c-MYC nuclear levels and correlates with differential expression of multiple genes, including ones involved in apoptosis. The ability of ectopically expressed Anks that carry an F-box to reproduce these phenomena supports that this strategy contributes to the microbe’s ability to inhibit apoptosis. Only a select few F-box-containing Anks that bind Cul1 exclude it from the nucleus. Hence, there are layers of complexity to these multifunctional effectors’ mechanisms that remain to be discerned. As inhibiting apoptosis is essential for maintaining an obligate intracellular microbial population, the mechanism reported herein is presumably one of many that *O. tsutsugamushi* employs.

## MATERIALS AND METHODS

### Cell cultivation and *O. tsutsugamushi* infection

Uninfected HeLa cells [CCL-2; American-type Culture Collection (ATCC), Manassas, VA] and RF/6A *Macaca mulatta* choroidal endothelial cells (CRL-1780; ATCC) were maintained as described (36, 43). *O. tsutsugamushi* str. Ikeda (NC_010793.1) was maintained in HeLa cells as described (36). For synchronous infection experiments, *O. tsutsugamushi*-infected HeLa cells (>90%) were mechanically disrupted using glass beads, followed by differential centrifugation to recover host cell-free bacteria as described (36), and then incubated with naïve HeLa or RF/6A cells at an MOI of approximately 10, unless stated otherwise. For mock infections, cells were incubated with an equivalent volume of bead-lysed host cells. At 2 to 4 h post-infection, an aliquot of the infected culture was fixed and permeabilized with ice-cold methanol while the remaining media was replaced with fresh media containing 1% (vol/vol) fetal bovine serum (Gemini BioSciences) to synchronize the infection. The collected sample was immunolabeled with antibody against *O. tsutsugamushi* TSA56 (18) and examined using immunofluorescence microscopy as previously described (36) to confirm that the desired MOI was achieved.

### *O. tsutsugamushi* Anks examined and bioinformatic analyses

Ectopically expressed versions of these Anks were used herein: Ank1_02 (OTT_RS03615), Ank2 (OTT_RS00235), Ank3_08 (OTT_RS05410), Ank4_01 (OTT_RS00980), Ank5_01 (OTT_RS01000), Ank6_02 (OTT_RS05585), Ank7_02 (OTT_RS09885), Ank8 (OTT_RS01225), Ank9 (OTT_RS01425), Ank10_01 (OTT_RS01880), Ank11 (OTT_RS02210), Ank12_01 (OTT_RS02915), Ank13 (OTT_RS04140), Ank14 (OTT_RS04940), Ank15 (OTT_RS05980), Ank16 (OTT_RS06155), Ank17 (OTT_RS07150), Ank19 (OTT_RS07355), and Ank20 (OTT_RS07600). For Anks that are followed by an underscore and a number, the number indicates the representative paralog of a multicopy paralogous family. Ank18 was excluded because it does not contain ARs, a PRANC, or F-box domain and merely consists of remnant sequence homologous to portions of other Anks.

### Plasmid constructs

pFlag-BAP was purchased from Sigma-Aldrich (St. Louis, MO). Constructs encoding mammalian codon-optimized Anks, Flag-Ank1-F-boxAAAAA, and Flag-Ank6-F-boxAAAAA N-terminally fused to the Flag tag or GFP were described previously (21, 25, 36).

### Transfection

HeLa cells grown to 80–90% confluency were transfected with plasmid DNA using Lipofectamine 2000 (Invitrogen, Carlsbad, CA) and incubated at 37°C in a humidified incubator at 5% atmospheric CO_2_ for 16 to 18 h. The amount of plasmid DNA used for transfections was modified from the Lipofectamine 2000 protocol to accommodate varying levels of transfection efficiency, as determined by western blot densitometric signal. In some cases, at 16 h post-transfection, transfected cells were treated with TNFα (25 ng mL^−1^) (Life Technologies, Grand Island, NY) or vehicle control [0.1% bovine serum albumin in H_2_O] at 37°C in a humidified incubator with 5% CO_2_ for 30 min prior to collection. After incubation, spent media was removed, and cells were washed with phosphate-buffered saline (PBS; 1.05  mM KH_2_PO4, 155  mM NaCl, 2.96  mM Na_2_HPO4, pH 7.4) before being processed for downstream applications.

### Nuclear fractionation and western blot

Transfected or *O. tsutsugamushi*-infected cells were washed with PBS, collected, and lysed following the Nuclear Fractionation Kit (Abcam, Cambridge, United Kingdom) protocol. In some cases, chloramphenicol was added to a final concentration of 34 µg mL^−1^ at 24 hpi, followed by collection at 72 hpi. Protein concentrations were determined by Bradford assay, and equivalent amounts of cytoplasmic and nuclear fractions were resolved by SDS-PAGE in 4 to 20% TGX polyacrylamide gels (Bio-Rad), blocked, immunolabeled, and imaged as previously described (17). Primary antibodies used for screening were rabbit or mouse anti-Flag [1:1,000; Sigma-Aldrich (F7425 or F1804)], rabbit anti-Cullin1 [Abcam (ab75817); 1:1,000], rabbit anti-Skp1 [Cell Signaling, Danvers, MA (2156S); 1:750], rabbit anti-Rbx1 [Abcam (ab133565); 1:1,000], rabbit anti-TSA56 (18), rabbit anti-NEDD8 [Abcam (ab81264); 1:1,000], rabbit anti-lamin A/C [Cell Signaling (2032S); 1:1,000], mouse anti-GAPDH [Santa Cruz, Dallas, TX (sc-365062); 1:750], mouse anti-Hsp90 [Santa Cruz (13119); 1:250], rabbit anti-p65 [Invitrogen (PA1-186); 1:1,000], and mouse anti-c-MYC [Santa Cruz (9E10); 1:200]. Bound primary antibodies were detected with horseradish peroxidase-conjugated horse anti-mouse or anti-rabbit IgG [Cell Signaling Technology (7076S or 7074S); 1:10,000]. Blots were incubated with SuperSignal West Pico PLUS, SuperSignal West Dura, or SuperSignal West Femto chemiluminescent substrate (Thermo Fisher Scientific) prior to imaging in a ChemiDoc Touch Imaging System (Bio-Rad). Bio-Rad Image Lab 6.0 software was used to obtain densitometric values.

### Immunoprecipitation

*O. tsutsugamushi*-infected and uninfected HeLa cells were harvested and lysed in high-saline Tris buffer (50 mM Tris HCl, 400 mM NaCl, 1 mM EDTA, pH 7.4) with 1.0% Triton X-100 (TBHS-T) spiked with Halt Protease and Phosphatase Inhibitor Cocktail (100×; Thermo). Protein A/G-coated agarose beads (Thermo Fisher Scientific) were washed with TBHS-T buffer two times, centrifuged at 8,600 × *g* for 1 min, and added to normalized cell lysates in a final volume of 400 µL. The samples were rotated with beads at 4°C for 4 h, followed by centrifugation at 8,600 × *g* for 1 min. Recovered supernatants were mixed with 1 µg of NEDD8 antibody and rotated at 4°C overnight. Washed protein A/G agarose beads were added, and the samples were rotated at 4°C for 1–2 h. Next, the samples were centrifuged at 8,600 × *g* for 1 min and washed with TBHS-T four times. Washed beads were resuspended in Laemmli buffer containing 5% β-mercaptoethanol and incubated at 100°C for 5 min to elute bound proteins. Inputs (30 µg) and eluates were resolved by SDS-PAGE and subjected to western blot analysis using Cul1, GAPDH, TSA56, and NEDD8 antibodies.

### RNAseq mining

Cross-referencing upregulated genes in *O. tsutsugamushi*-infected HeLa cells at 48 h identified by our RNAseq analysis (17) with the list of 2704 Cul1-regulated genes in HeLa cells determined by Sweeney et al (38) identified 107 shared genes (Data Set S1). GO enrichment analysis of all upregulated genes at 48 hpi was performed using the DESeq2 R software package 1.14.1 (Bioconducter) (17, 69). GO terms with a significant false discovery rate (*P* < 0.05) were assessed for the inclusion of one or more of the 107 genes. GO biological processes that contained at least six of the overlapping genes and were associated with cell proliferation, signal transduction, and apoptosis were prioritized (Table 1 and 2). Twenty-one genes within these prioritized GO terms were selected for further analysis by RT-qPCR (Table 2 and 3).

**TABLE 3:** Oligonucleotides used in this study

Oligonucleotide	Sequence (5′ to 3′)
ank1 191F	CTGCTTATAACGGCAACATAC
ank1 289R	CAATGTTACTTTGTTCAACAGC
ank6 178F	GCGTTACATGTTGCTTCTTAC
ank6 304R	CAGAAGACGTACAATATCAATGTG
ott16S 911F	GTGGAGCATGCGGTTTAATTCGATGATC
ott16S 1096R	TAAGAATAAGGGTTGCGCTCGTTGC
GAPDH 8182F	ACATCATCCCTGCCTCTACTGG
GAPDH 8351R	TCCGACGCCTGCTTCACC
ADAM19 1128F	ATGGGCCACAACTTTGGCAT
ADAM19 1279R	CACATTCCACCACCTGACT
CDK6 4555F	TCCCTCCTTTGAAGTGGATG
CDK6 4684R	GTCACCTGGGGCTAAATGAA
KLF5 1148F	CCACCACCCTGCCAGTTAAC
KLF5 1286R	TAAACTTTTGTGCAACCAGGGTAA
WDR3 86F	ACCAAGCAGTACCTACGCTAT
WDR3 209R	TAAGTCCCAGATGAAAACGTGTTC
KANK1 2455F	AACAGGCAGCAA CACAGAGGAG
KANK1 2523R	CACCTGGCTAACAGCCT
DUSP10 12F	CGTCACCACTCATCTTCCCC
DUSP10 170R	ACACTGGTGAGCTTCCTCAAT
CNOT6L 1161F	TTCTGATGTGAAGCTCATCCAG
CNOT6L 1361R	TCATTGTACCTTAGTTCCTTGAAGT
PLK2 506F	ATTGACAAAGAAATAGAGCTTCACAG
PLK2 674R	TCTGCCTGAGGTAGTATCG
VEGFC 1076F	ATGTGTGTCCGTCTACAGATGT
VEGFC 1213R	GGAAGTGTGATTGGCAAAACTGA
NR3C1 2670F	AGTGGTTGAAAATCTCCTTAACTATTGCT
NR3C1 2747R	GGTATCTGATTGGTGATGATTTCAGCTA
WDR43 379F	GGTAGCATTTTATTATACAGCACAGTAAAAGGAGAGTT
WDR43 497R	CCTTTCCATTTGCACTTTACTTTGCATGTCTGT
UTP25 1230F	AGCAAGAAGAAAATCATTGTGAGC
UTP25 1394R	GGCATAGAGTCGGATGCTT
ITPR1 129F	TTCACATCTCAGAACTAGCCACA
ITPR1 276R	GGGTCACTGCCTAACTCATTC
NRIP1 1908F	ATTCCAACTGTGTTCCCATAGA
NRIP1 1997R	CCCAAGTGTTTAGCAAGGATTG
MCL1 7F	TGGCGGAAGCGCCGGCGC
MCL1 146R	TTCCGAAGCATGCCTTGG
PMAIP1 179F	GGAGATGCCTGGGAAGAAG
PMAIP1 251R	CCTGAGTTGAGTAGCACACTCG
HERC6 597F	CCACTCCCTGGCATTATCAAAA
HERC6 776R	GCCAAACGAAGTCCCACAGA
ABL2 2643F	GTGATGAGACTACTGCAGCATCC
ABL2 2801R	GCACTTGAGGTGGAGGC
PAK1IP1 529F	GGTCCCCAAGAGGAGAG
PAK1IP1 690R	AGTGAATCACAGTCAAAAAACCTTATAAC
URB2 4444F	GAGTTTGCTGTGTTTTCCCC
URB2 4542R	GAGGTCCAGGATGAGGTAAATG
TTL 1062F	AAGGAACTGCCTCCTGAGC
TTL 1163R	TCAATGAGCCAC ACCTTCA


### RT-qPCR

Total RNA isolated using the RNeasy Mini Kit (Qiagen) was eluted in 25 µL of RNase-free water, followed by concentration and purity determination. Subsequently, 1 μg of RNA was treated with amplification-grade DNase I (Invitrogen) per the manufacturer’s protocol. cDNA was generated using Bio-Rad’s iScript Reverse Transcription Supermix protocol. Parallel reactions were performed in the absence of reverse transcriptase. To confirm genomic DNA depletion, both samples were subjected to PCR with human *GAPDH*-specific primers (41) and MyTaq polymerase (Bioline, Taunton, MA). The amplicons were visualized by agarose gel electrophoresis. qPCR using the cDNA as template was performed with SsoFast EvaGreen supermix (Bio-Rad) and primer pairs for *O. tsutsugamushi* 16S rDNA (*ott16S*) (42), human *GAPDH* (41), and human genes of interest listed in Table 3. Thermal cycling conditions used were 95°C for 30 s, followed by 40 cycles of 95°C for 5 s and 55°C for 5 s, followed by a melt curve from 60 to 95 °C. Relative expression was determined using the 2^−ΔΔCT^ method (70) as part of the CFX Maestro for Mac 1.0 software package (Bio-Rad).

### Bioinformatic analyses

CLUSTALΩ (https://www.ebi.ac.uk/Tools/msa/clustalo/) (71) was used to generate an alignment of the AR regions of each Ank analyzed herein as defined by Beyer et al.: Ank1_02 residues 23–118, Ank2 1–134, Ank3_08 6–182, Ank4_01 50–315, Ank5_01 2–132, Ank6_02 23–158, Ank7_02 35–129, Ank8 1–173, Ank9 22–256, Ank10_01 20–319, Ank11 10–161, Ank12_01 35–281, Ank13 18–285, Ank14 33–193, Ank15 105–195, Ank16 5–125, Ank17 37–129, Ank19 4–163, and Ank20 32–335 (23). The alignment was then analyzed using Interactive Tree of Life (iTOL) v4 (https://itol.embl.de/) (72) to construct a phylogenetic tree. STRING (50) was used to analyze interaction networks of Cul1-regulated genes exhibiting a significantly different expression in *O. tsutsugamushi*-infected cells.

### Flow cytometry

Uninfected or *O. tsutsugamushi*-infected cells or cells expressing GFP and GFP-Anks were treated with a vehicle or staurosporine 24 h prior to collection. At the time of collection, samples were washed with PBS, dislodged with 0.526 mM EDTA (EDTA) solution, and pelleted at 1000 × *g* for 3 min. The pellet was resuspended in 1× annexin V binding buffer [0.1 M HEPES (pH 7.4), 1.4 M NaCl, and 25 mM CaCl_2_], and uninfected or infected samples were incubated with FITC–annexin V and propidium iodine (PI), while cells expressing GFP or GFP-Anks were incubated with PE–annexin V and 7-AAD. Samples were incubated in the dark at room temperature for 15 min. Prior to assessment by flow cytometry, samples were diluted in 1× annexin V binding buffer. Single cell-gated populations were quantified for 10,000 uninfected and infected cells, and 10,000 cells gated for GFP-positivity were enumerated in cells expressing GFP or GFP-Anks on a BD FACSMelody with BDFACSChorus 1.3.3 software (BD Biosciences). Compensation and quadrant analysis were performed using FlowJo software (version 10.8.1; BD Biosciences). Cells in quadrant 1 (Q1; PI+/FITC− or 7-AAD+/PE−) and Q2 (PI+/FITC+ or 7-AAD+/PE+) were considered late apoptotic/dead. Cells in Q3 (PI−/FITC + or 7-AAD−/PE+) were deemed early apoptotic, while those in Q4 (PI−/FITC− or 7-AAD−/PE−) were considered live. The fold change in % late apoptotic/dead cells was calculated by subtracting the % late apoptotic/dead cells of each vehicle control-treated sample from that of its staurosporine-treated counterpart and dividing the difference by its respective vehicle control.

### Statistical analyses

Statistical analyses were performed using the Prism 8.0 software package (GraphPad, San Diego, CA). One-way analysis of variance (ANOVA) with Tukey’s or Dunnett’s *post hoc* tests was used to test for a significant difference among groups. Student’s *t*-test was performed to test for a significant difference among pairs. Statistical significance was set at *P* values of < 0.05.

## SUPPLEMENTAL MATERIAL

Data S1 iai.00559-24-s0001.xlsx Cul1 target genes that are significantly upregulated in *O. tsutsugamushi*-infected HeLa cells at 48hpi and GO biological processes associated with upregulated genes in cells infected with *O. tsutsugamushi* and Cul1 regulated genes that are within them.

Supplemental material iai.00559-24-s0002.docx References for supplemental data.

ASM does not own the copyrights to Supplemental Material that may be linked to, or accessed through, an article. The authors have granted ASM a non-exclusive, world-wide license to publish the Supplemental Material files. Please contact the corresponding author directly for reuse.
