# Supplementary material for: Orientia tsutsugamushi alters the intranuclear balance of cullin-1 and c-MYC to inhibit apoptosis
Source: Infect Immun. 2025 Feb 20;93(3):e00559-24. doi: 10.1128/iai.00559-24 (PMC11895443; doi:10.1128/iai.00559-24)
Supplement: Supplemental material — References for supplemental data. [file iai.00559-24-s0002.docx]

**SUPPLEMENTAL REFERENCES**

1. Sweeney MA, Iakova P, Maneix L, Shih FY, Cho HE, Sahin E, Catic A. 2020. The ubiquitin ligase Cullin-1 associates with chromatin and regulates transcription of specific c-MYC target genes. Sci Rep 10:13942.
2. Adcox HE, Hatke AL, Andersen SE, Gupta S, Otto NB, Weber MM, Marconi RT, Carlyon JA. 2021. Orientia tsutsugamushi Nucleomodulin Ank13 Exploits the RaDAR Nuclear Import Pathway To Modulate Host Cell Transcription. mBio doi:10.1128/mBio.01816-21:e0181621.
